# Dynamics and immunological signature of γδ T cells following antiretroviral therapy initiation in acute HIV-1 Infection

**DOI:** 10.3389/fimmu.2025.1554916

**Published:** 2025-05-08

**Authors:** Haihan Wang, Sibo Li, Rui Wang, Xia Wang, Yang Zhang, Xiaofan Lu, Jianping Sun, Tong Zhang, Xiaojie Huang, Bin Su, Hao Wu, Zhen Li

**Affiliations:** ^1^ Beijing Key Laboratory for HIV/AIDS Research, Beijing Youan Hospital, Capital Medical University, Beijing, China; ^2^ Clinical Research Center of Infectious Diseases, Beijing Youan Hospital, Capital Medical University, Beijing, China; ^3^ Biomedical Information Center, Beijing Youan Hospital, Capital Medical University, Beijing, China

**Keywords:** γδ T cells, early/acute HIV-1 infection, antiretroviral therapy (ART), immune activation, regulatory T cells

## Abstract

Early antiretroviral therapy (ART) is essential for controlling HIV-1 replication and boosting immune function. γδ T cells, as a vital component of the innate immune system, are implicated in the antiviral response. However, their immunological profile during acute HIV-1 infection and the early stages of ART remains unclear. This study aimed to delineate the immunological landscape of γδ T cells in individuals with acute HIV-1 infection undergoing early ART. We enrolled 65 participants who initiated ART immediately post-diagnosis and assessed the phenotypes and functions of γδ T cells using flow cytometry. We demonstrated that early ART significantly increased the frequency of Vδ2 T cells, while the Vδ1 T cell frequency remained stable and showed an inverse relationship with CD4^+^ T cell counts after ART. Early ART normalized the activation and PD-1 expression in Vδ1 and Vδ2 T cells, aligning with healthy controls (HCs) levels. Nevertheless, the proliferation of these cells, particularly within the PD-1^+^ subset, remains elevated post-ART. We also noted a reduction in perforin secretion in PD-1^+^ Vδ1 and Vδ2 T cells of people living with HIV (PLWH). Furthermore, Vδ1 T cells were identified as the predominant regulatory T cells, with TGF-β production and co-expression of CD127 and CXCR4, negatively correlated with CD8^+^ T cell activation. Our study elucidates the dynamic immunological characteristics of γδ T cells in acute HIV-1 infection and early ART, contributing to the understanding of their role in HIV-1 pathogenesis and the potential for γδ T cell-based immunotherapeutic strategies.

## Introduction

Human immunodeficiency virus-1 (HIV-1) infection remains a significant global health challenge, with the absence of a universally effective vaccine and a definitive cure (1). The advent of ART has transformed HIV-1 infection into a manageable chronic condition, playing a crucial role in mitigating immune activation and inflammation, and restoring immune function. Despite these advances, T cell dysfunction persists, hindering immune reconstitution in people living with HIV-1 (PLWH) (2, 3).

The acute phase of HIV-1 infection is critical, influencing disease progression, with innate immune response, particularly γδ T cells, playing a crucial role in controlling viral spread (4). Intense immune activation is a hallmark of acute HIV-1 infection. Early initiation of ART during this phase not only attenuates soluble immune activation markers but also restores T cell activation levels to those observed in healthy controls (5, 6). However, early ART does not completely restore T cell functionality, as our previous study have demonstrated that sustained T cell proliferation in individuals with acute HIV-1 infection despite early ART, especially in those who are immunological non-responders (7).

γδ T cells, a minor subset of the T cell repertoire in the peripheral blood of healthy adults, bridge innate and adaptive immune responses, defending against neoplastic and pathogenic challenges (8, 9). γδ T cells are categorized into Vδ1 and Vδ2 subsets. Vδ1 T cells are predominantly localized in mucosa-associated lymphoid tissues, such as intestine and lung, where they recognize stress-induced antigens, and regulate immune responses and maintain tissue homeostasis. While Vδ2 T cells, enriched in peripheral blood and lymph node, and respond to phosphoantigens. They secrete anti-viral factors and cytokines, and are capable of lysing tumor or infected cells, contributing to immune surveillance and control of pathogen invasion (10).

HIV-1 infection disrupts the balance of γδ T cell subsets, reducing Vδ2 T cell numbers and increasing Vδ1 T cell counts, that accelerate the disease progression (11, 12). In chronic HIV-1 infection, γδ T cells are anergic, exhibiting a diminished response to isopyrophosphate (IPP) and reduced cytotoxicity. Moreover, HIV-1 infection leads to γδ T cell over-activation and exhaustion, that only partially mitigated by late-stage ART initiation (11). Our previous work has shown a gradual decline in γδ T cell cytotoxicity during acute HIV-1 infection over time (13). However, the immunological dynamic of γδ T cells during acute HIV-1 infection and the impact of early ART on their phenotypes and functions are not well understood.

In the present study, we investigated the effect of early ART on the immunological landscape of γδ T cells in individuals with acute HIV-1 infection. We find that early ART increases the frequency of Vδ2 T cells without significantly altering Vδ1 T cells, which inversely correlate with CD4^+^ T cell counts. Early ART also normalizes the activation status and PD-1 expression levels in both Vδ1 and Vδ2 subsets, although proliferation of these cells, particularly within the PD-1^+^ subset, remains elevated. Furthermore, we identify Vδ1 T cells as a major component of regulatory T cells, with elevated TGF-β production and co-expression of CD127 and CXCR4. These findings provide shed light on the dynamic immunological characteristics of γδ T cells in the context of acute HIV-1 infection under early ART, offering insights into potential γδ T cell-based immunotherapeutic strategies.

## Materials and methods

### Subjects

We conducted a prospective study enrolling a total of sixty-five people living with acute HIV-1 infection (PLWAHs) from the clinical research cohort of Beijing PRIMO (14, 15). Acute HIV-1 infection was defined as positive for HIV-1 RNA and negative or indeterminate for anti-HIV-1 antibodies. The inclusion criteria for PLWAHs were male gender, an age range from 18 to 60 years, and no history of prior ART treatment. Exclusion criteria were encompassed individuals co-infected with hepatitis B virus, hepatitis C virus, or tuberculosis. As a comparator group, we enrolled age-matched HIV-1-negative men who have sex with men (MSM), serving as healthy controls (HCs, n = 21).

PLWAHs were promptly initiated on ART upon definitive diagnosis, and subsequently underwent follow-up assessments at weeks 24, 48, and 96. Venous blood samples were collected at baseline and during each follow-up visit for the isolation and cryopreservation of peripheral blood mononuclear cells (PBMCs) in liquid nitrogen for subsequent analysis. The study protocol was approved, and written informed consent was obtained from all participants in compliance with the ethical standards outlined in the Declaration of Helsinki. This study was approved by the Beijing Youan Hospital Research Ethics Committee (No.2024-072, approved date: 2024-03-11).

### Determination of absolute CD4^+^ T cell count

The whole blood CD4^+^ T-cell absolute count of each sample was determined with a TruCount tube with multicolor antibodies (anti-human CD3, CD45, CD4, and CD8 cell markers), performed by BD Canto II flow cytometer (BD Biosciences, New Jersey, USA).

### Plasma HIV-1 RNA quantification

The plasma HIV-1 viral load was quantified using the automated Real-time PCR M2000 system (Abbott Molecular Inc., Des Plaines, IL, USA), with 40 copies/mL detection limitations.

### Antibodies

FITC-conjugated anti-human Vδ1TCR (TS8.2) mAb, Peridinin-Chlorophyll-Protein Complex-cyanine5.5 (Percp-cy5.5)-conjugated anti-human Vδ2TCR (B6) mAb, Phycoerythrin-cyanine7 (PE-cy7)-conjugated anti-human CD3 (OKT3) mAb, Phycoerythrin (PE)-conjugated anti-human CD38 (HIT2), Allophycocyanin (APC)-conjugated HLA-DR (L243) mAb, PE-conjugated anti-human Vδ2TCR (B6) mAb, Allophycocyanin-cyanine 7 (APC-cy7)-conjugated anti-human CD3 (OKT3) mAb, APC-cy7-conjugated anti-human CD4 (RPA-T4) mAb, Percp-cy5.5-conjugated CD56 (5.1H11) mAb, Brilliant violent (BV) 421-conjugated anti-human PD-1 (NAT105) mAb, BV421-conjugated anti-human CD8 (SK1) mAb, BV510-conjugated anti-human CXCR4 (12G5) mAb, APC-cy7-conjugated anti-human CD127 (A019D5) mAb, PE-cy7-conjugated anti-human Ki67 (Ki-67) mAb, APC-conjugated anti-human Perforin (B-D48) mAb, Pacific Blue-conjugated anti-human Foxp3 (206D) mAb, and PE-conjugated anti-human TGF-β1 (S20006A) mAb. Live/dead fixable viability stain (FSV) 510 was also used. All the fluorescent antibodies and isotype controls were purchased from ThermoFisher, Biolegend and BD biosciences.

### Cell staining and flow cytometry

Cryopreserved PBMCs were rapidly thawed and washed with phosphate-buffered saline (PBS) supplemented with 1% bovine serum albumin (BSA). Subsequently, cells were labeled with surface-specific antibodies, and incubated at room temperature (RT) for 20 min in the dark to prevent photobleaching. After surface staining, cells were fixed and permeabilized for intracellular staining, followed by labeling with intracellular or nuclear antibodies, and incubated at RT for 30 min in darkness. Post-incubation, cells were washed, and fixed with 2% paraformaldehyde (PFA). A minimum of 200,000 lymphocytes were acquired using a BD FACS Canto II flow cytometer. Data analysis was conducted using FlowJo software (version 10.9).

### Statistical analysis

Data were presented as the median and interquartile range (IQR). SPSS (21.0) and GraphPad Prism 9.3 were used for statistical analysis and figure creation. Differences in the parameters between HCs and PLWAHs were calculated using the Manny-Whitney *U* test. Differences in the parameters among HCs and PLWAHs at each time point were calculated using the Kruskal-Wallis test, and differences in the parameters between baseline and follow-ups were assessed by the Friedman test or the Wilcoxon matched-pairs signed rank test. Correlations were performed by using the non-parametric Spearman’s rank correlation test. All the tests were two-tailed, and *P* values less than 0.05 were considered statistically significant.

## Results

### Clinical characteristics of the participants

Description of the clinical information of all participants is presented in **Table 1**. The study cohort consisted exclusively of male individuals, with no significant difference in age between PLWAHs and HCs (*P* = 0.35). Among the PLWAHs, the median estimated time since infection was 66 days, ranging from 36 to 98 days. 15.4% of them were in the early Fiebig stage I/II, while 84.6% of them were in the late Fiebig stage V/VI. At baseline, the median CD4^+^ T-cell counts was 376 cells/μL, which increased significantly to 552 cells/μL at 24 weeks, 578 cells/μL at 48 weeks, and 653 cells/μL at 96 weeks following ART initiation. Concurrently, the median CD4/CD8 ratio improved from 0.39 at baseline to 0.88 after 96 weeks of ART. Plasma HIV-1 RNA levels, with a median of 4.4 Log_10_ copies/mL at baseline, were undetectable (< 40 copies/mL) in all participants after 96 weeks of ART.

**Table 1:** Basic characteristics of all the participants.

Characteristics	HCs	PLWAHs	*P* values
Cases	21	65	
Age (y)	28 (25 - 46)	31 (18 - 52)	0.35
Infection time (day)	NA	66 (36 - 98)	
Fiebig stage	NA		
I		7 (10.7%)	
II		3 (4.7%)	
V–VI		55 (84.6%)	
CD4^+^ T cell Counts at baseline (cells/µL)	NA	376 (282 - 498)	
CD4^+^ T cell Counts at 24 weeks (cells/µL)	NA	552 (403 - 672) ^a^	*P* < 0.001
CD4^+^ T cell Counts at 48 weeks (cells/µL)	NA	578 (438 - 707) ^b^	*P* < 0.001
CD4^+^ T cell Counts at 96 weeks (cells/µL)	NA	653 (545 - 790) ^c^	*P* < 0.001
CD4/CD8 ratio at baseline	NA	0.39 (0.26 - 0.63)	
CD4/CD8 ratio at 96 weeks		0.88 (0.62 - 1.16) ^d^	*P* < 0.001
Plasma viral load (log_10_ copies/mL)	NA	4.4 (3.9 - 5.1)	
Drugs	NA		
TDF+3TC+EFV		62 (95%)	
TDF+3TC+LPV/r		3 (5%)	

Data were depicted as median and IQR (interquartile range). *P* values were calculated by Mann-Whitney *U* test and Wilcoxon matched-pairs test. NA, not applicable. a, b, c, d, compared with baseline, *P* < 0.001.

### Dynamics of γδ T cell subsets in PLWAHs post-early ART

**Figure 1:**
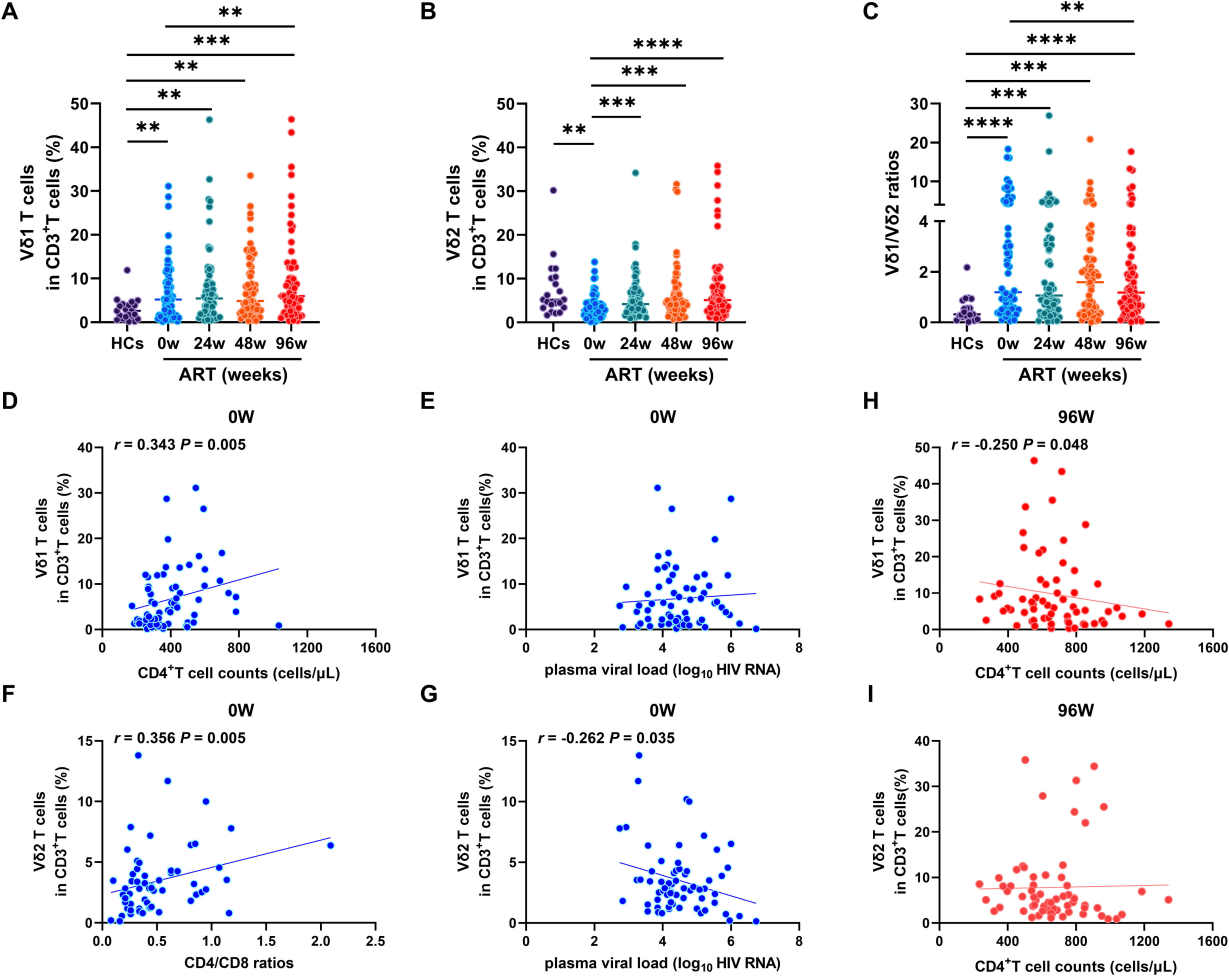
Dynamic of γδ T cell subsets after early ART. Comparison of the frequencies of Vδ1 T cells **(A)**, Vδ2 T cells **(B)** and Vδ1/Vδ2 ratios **(C)** among HCs, PLWAHs at baseline, week 24, 48 and 96 of early ART. Correlations of Vδ1 T cells with CD4^+^T cell counts **(D)** and plasma viral load **(E)** in PLWAHs at baseline. Correlations of Vδ2 T cells with CD4/CD8 ratios **(F)** and plasma viral load **(G)** in PLWAHs at baseline. **(H, I)** Correlation of Vδ1 T cells or Vδ2 T cells with CD4^+^T cell counts in PLWAHs after 96 weeks of ART. HCs, healthy controls; PLWAHs, people living with acute HIV-1 infection; **P < 0.01; ***P < 0.001; ****P < 0.0001.

We utilized flow cytometry to quantify the frequencies of Vδ1 and Vδ2 T cells within CD3^+^T cell compartment. We revealed that the frequency of Vδ1 T cells was significantly elevated in PLWAHs at baseline (week 0), and this increase was maintained through 24, 48 and 96 weeks of ART. Notably, the frequency of Vδ1 T cells remained higher than baseline levels even after 96 weeks of ART (**Figure 1A**). In contrast, the frequency of Vδ2 T cells was markedly reduced in PLWAHs at baseline, but we observed a gradual increase in their frequency following early ART, particularly after 24 weeks (**Figure 1B**). Moreover, the Vδ1/Vδ2 ratio was significantly higher in PLWAHs at all time points compared to HCs, and this ratio decreased after 96weeks of ART to levels lower than those at baseline (**Figure 1C**).

We further explored the correlation between the frequencies of Vδ1 and Vδ2 T cells and various clinical parameters within the PLWAH cohort. We detected a positive correlation between the frequency of Vδ1 T cells and absolute CD4^+^ T cell counts at baseline (**Figure 1D**), yet no significant association with plasma viral load was observed (**Figure 1E**). Significantly, we found a positive correlation between the frequency of Vδ2 T cells and CD4/CD8 ratios (**Figure 1F**), along with a negative association with plasma viral load at baseline (**Figure 1G**). Furthermore, after 96 weeks of ART, the frequency of Vδ1 T cells, but not Vδ2 T cells, was negatively associated with CD4^+^ T cell counts (**Figures 1H, I**). Taken together, these findings suggest that the imbalance of Vδ1 and Vδ2 T cells in acute HIV-1 infection is implicated in disease progression and affects immune reconstitution after early ART.

### Analysis of the phenotypes of γδ T cells

**Figure 2:**
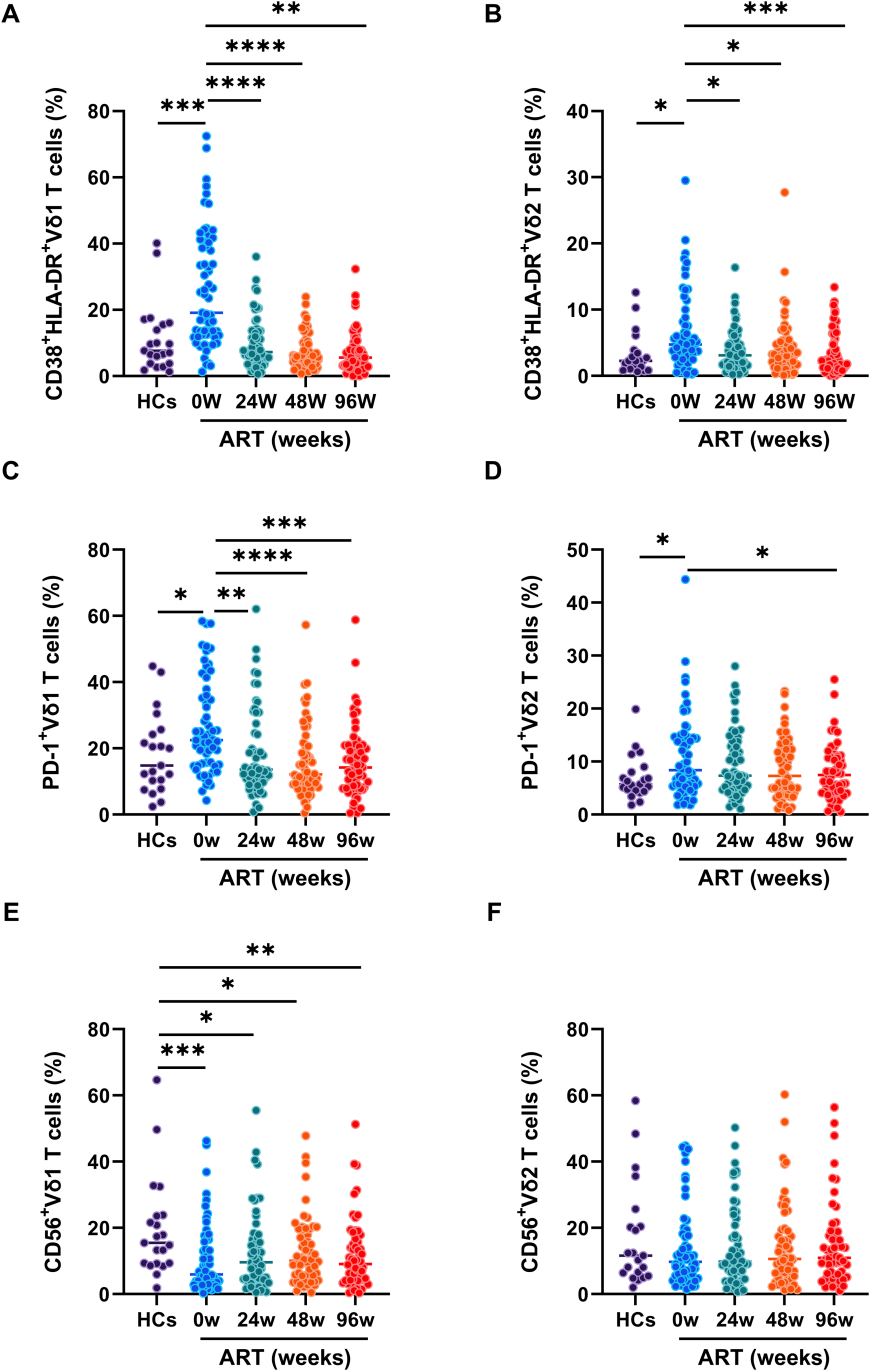
The phenotypes of γδ T cell subsets in PLWAHs. Markers for immune activation, exhaustion, and cytolysis on γδ T cell subsets were detected by flow cytometry. The frequencies of CD38^+^HLA-DR^+^Vδ1 T cells **(A)**, CD38^+^HLA-DR^+^Vδ2 T cells **(B)**, PD-1^+^Vδ1 T cells **(C)**, PD-1^+^Vδ2 T cells **(D)**, CD56^+^Vδ1 T cells **(E)**, CD56^+^Vδ2 T cells **(F)** were compared among HCs, PLWAHs at baseline, week 24, 48 and 96 of early ART. *P < 0.05; **P < 0.01; ***P < 0.001; ****P < 0.0001.

To investigate the exact role of γδ T cell subsets in acute HIV-1 infection, we measured the phenotypes of immune activation, exhaustion, and cytotoxicity related markers on Vδ1 and Vδ2 T cells. Compared to HCs, significant higher frequencies of CD38^+^HLA-DR^+^Vδ1 T cells and CD38^+^HLA-DR^+^Vδ2 T cells were observed in PLAWHs at baseline, and which were successfully decreased by early ART after 24 weeks (**Figures 2A, B**). Similarly, the frequencies of PD-1^+^Vδ1 T cells and PD-1^+^Vδ2 T cells were significantly increased in PLAWHs at baseline compared with HCs. After 96 weeks of early ART, the frequencies of these cells were decreased, and close to the levels of HCs (**Figures 2C, D**). Moreover, we found a remarkable decrease of the frequencies of CD56^+^Vδ1 T cells in PLAWHs at baseline than in HCs. These cells were still significantly lower than HCs even after early ART at week 24, 48 and 96 (**Figure 2E**). However, there were no statistical differences in the frequencies of CD56^+^Vδ2 T cells among HCs and PLAWHs at week 0, 24, 48 and 96 (**Figure 2F**).

### Early ART is inadequate for complete recovery of γδ T cell proliferation

**Figure 3:**
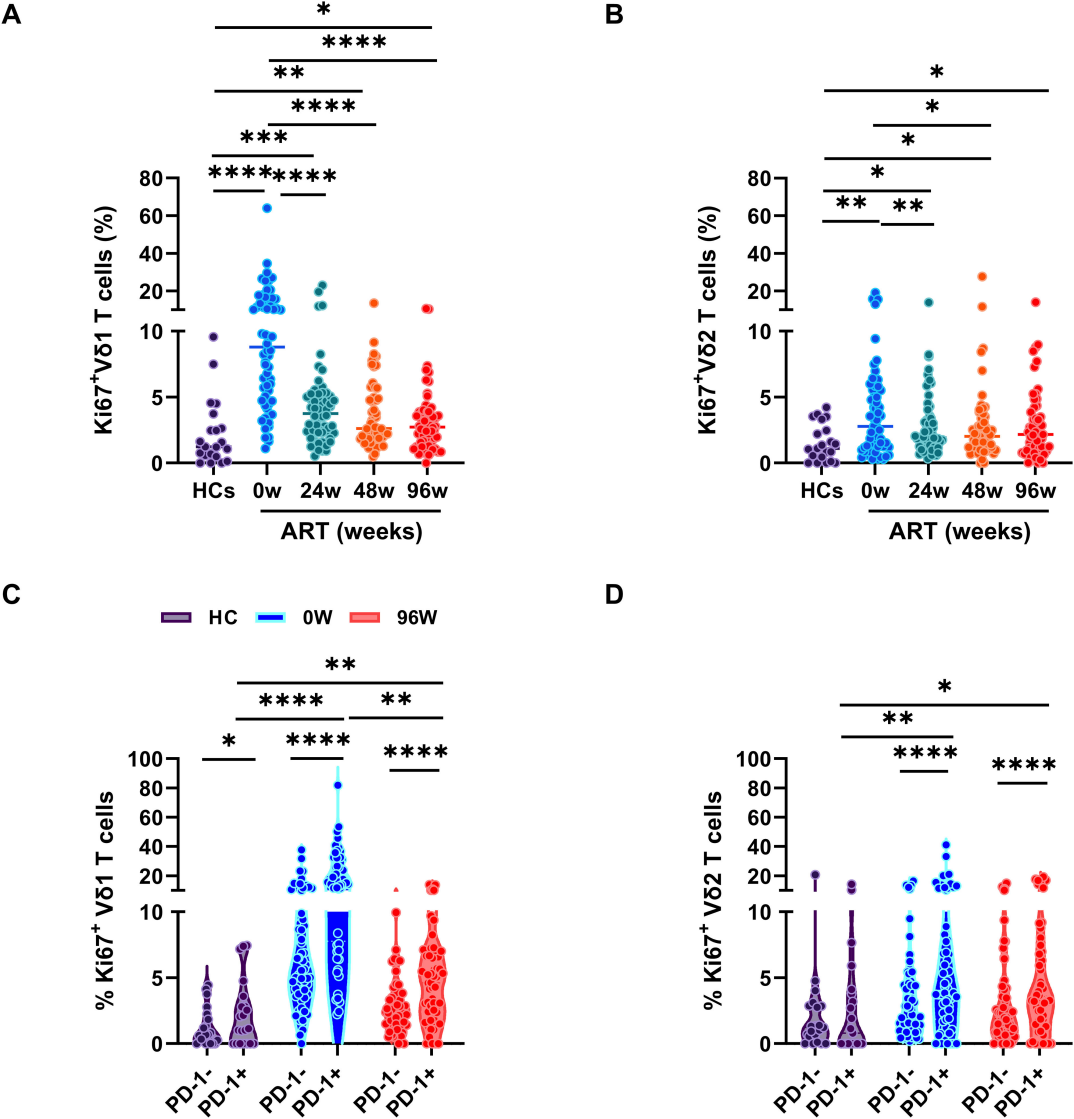
Early ART insufficient for full γδ T cell proliferation recovery. Comparison of the frequencies of Ki67 expression in Vδ1 T cells **(A)** or Vδ2 T cells **(B)** among HCs and PLWAHs at different timepoints. **(C, D)** Percentage of Ki67 expression in PD-1^+^ and PD-1^-^ Vδ1 T cells or Vδ2 T cells in HCs, PLWAHs at baseline and after 96 weeks of early ART. *P < 0.05; **P < 0.01; ***P < 0.001; ****P < 0.0001.

γδ T cells exert their immune response to pathogens through rapid activation and proliferation. In our investigation, we focused on the expression of Ki67, a protein that accumulates in the cytoplasm and nucleus during the active phases of the cell cycle, within distinct subsets of γδ T cells. Our results demonstrate a significant increase in Ki67 expression within both Vδ1 and Vδ2 T cell subsets in PLWAHs compared to HCs, with Vδ1 T cells showing a more pronounced increase at baseline (**Figures 3A, B**). Furthermore, early ART intervention significantly curtailed the prevalence of Ki67-positive Vδ1 and Vδ2 T cells. Nonetheless, even after 96 weeks of ART, the frequency of these cells remained higher in PLWAHs than in HCs (**Figures 3A, B**).

We further investigated the phenotypic characteristics of Ki67-positive cells within the Vδ1 and Vδ2 T cell subsets and discovered that these cells exhibit elevated levels of PD-1 expression, particularly in Vδ1 T cells from PLWAHs at baseline (**Figures 3C, D**). Notably, early initiation of ART was effective in normalizing PD-1 expression levels (**Figures 2C, D**) and in reducing the frequency of Ki67^+^PD-1^+^ Vδ1 and Vδ2 T cells. However, even after 96 weeks of ART treatment, a persistently higher prevalence of these dual-positive cells was observed in comparison to HCs (**Figures 3C, D**).

### The cytotoxicity of γδ T cell subsets

**Figure 4:**
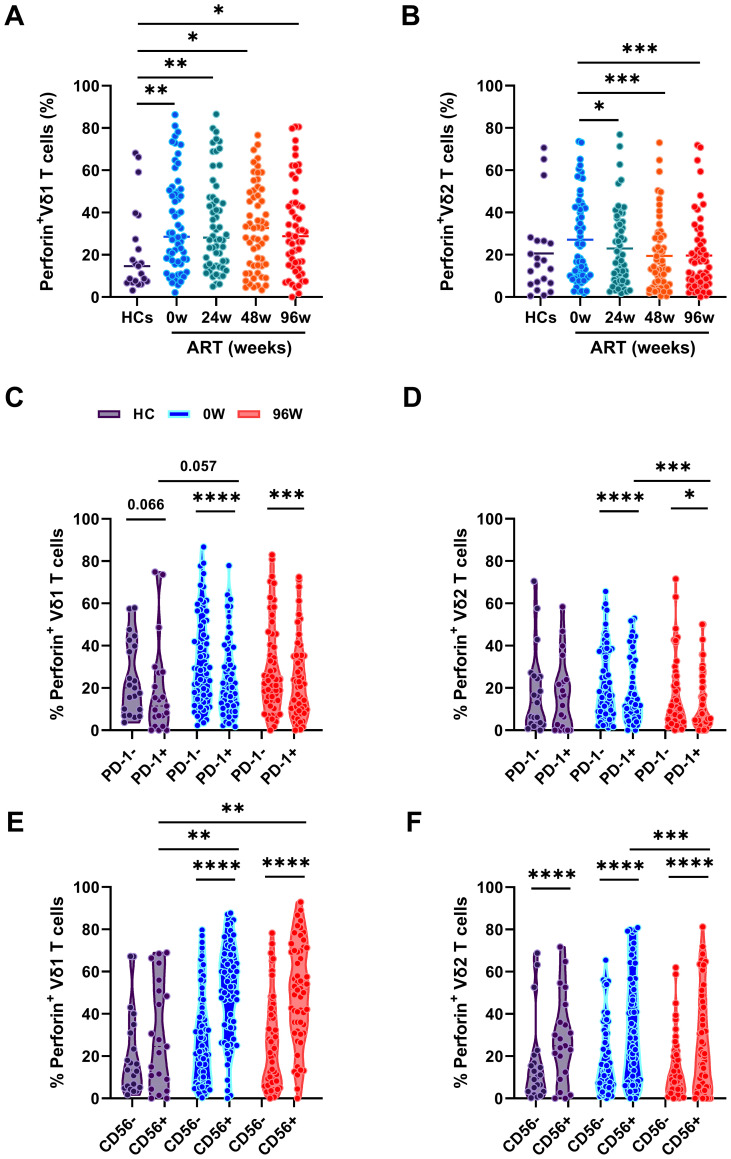
The cytotoxicity of γδ T cells. Comparison of the frequencies of perforin expression in Vδ1 T cells **(A)** or Vδ2 T cells **(B)** among HCs and PLWAHs at different timepoints. **(C, D)** Percentage of perforin expression in PD-1^+^ and PD-1^-^ Vδ1 T cells or Vδ2 T cells among HCs, PLWAHs at baseline and after 96 weeks of early ART. **(E, F)** Percentage of perforin expression in CD56^+^ and CD56^-^ Vδ1 T cells or Vδ2 T cells among HCs, PLWAHs at week 0 and week 96 after early ART. *P < 0.05; **P < 0.01; ***P < 0.001; ****P < 0.0001.

γδ T cells display critical role in fighting against HIV-1 infection, by directly killing HIV-1-infected CD4^+^T cells through the expression of cytotoxic molecules, such as perforin (16). To evaluate the kill ability of γδ T cell after early ART, we measured the perforin expression in both Vδ1 and Vδ2 T cells. We found that perforin expression in Vδ1 T cells was significantly higher in PLWAHs at week 0, and week 24, 48 and 96 after early ART compared with HCs, indicates that early ART fails to restore the cytotoxicity of Vδ1 T cells (**Figure 4A**). In contrast, the perforin expression in Vδ2 T cells were not significantly increase in PLWAHs at week 0, however, early ART reduced the expression of perforin in Vδ2 T cells (**Figure 4B**).

Next, we linked the cytotoxicity potential of γδ T cells with the phenotypic markers and found that the expression of perforin in PD-1^+^Vδ1 and Vδ2 T cells was markedly lower than in their PD-1^-^ counterparts in PLWAHs, both at baseline and after 96 weeks of early ART. In contrast, no statistically significant differences in perforin expression were observed between these subsets in HCs (**Figures 4C, D**). Furthermore, we noted a higher expression of perforin in CD56^+^Vδ1 and Vδ2 T cells compared to CD56^-^Vδ1 and Vδ2 T cells, a pattern that was present in PLWAHs at baseline and persisted after 96 weeks of ART, as shown in **Figures 4E, F**.

### Regulatory γδ T cells in early HIV-1 infection

**Figure 5:**
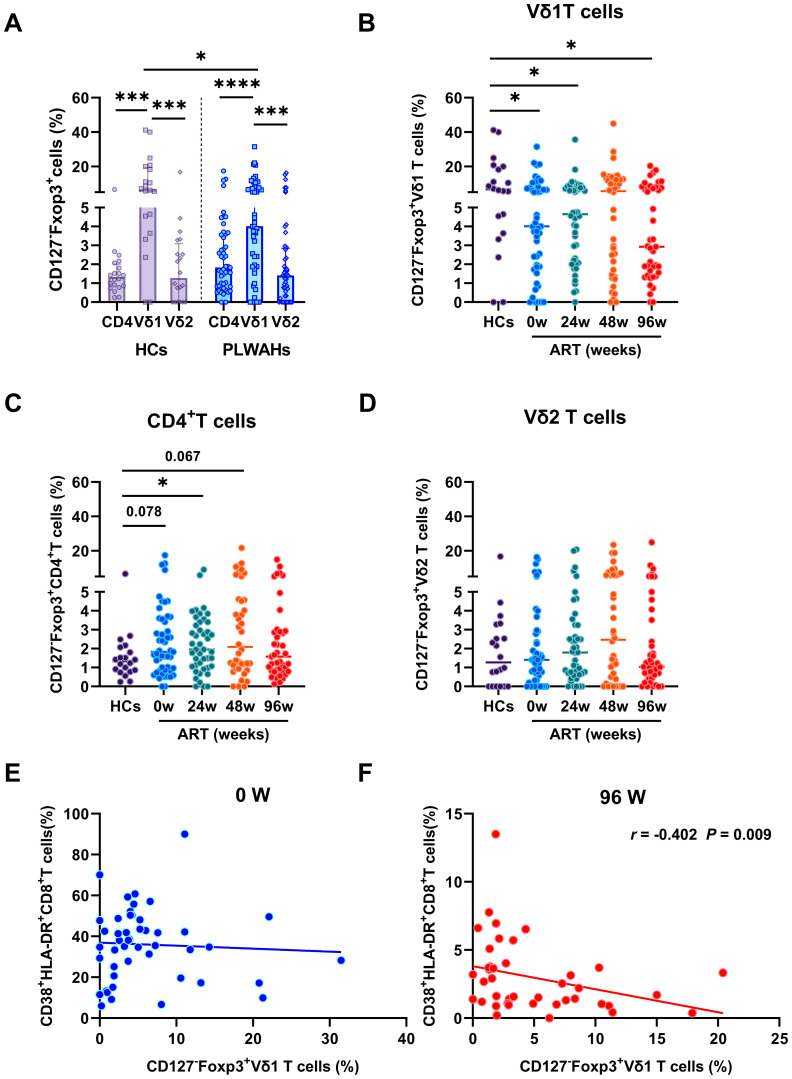
Dynamic of regulatory γδ T cells in early HIV-1 infection. **(A)** The frequencies of Tregs in CD4^+^T cells, Vδ1 and Vδ2 T cells in HCs and PLWAHs. **(B-D)** Comparisons of the frequencies of Tregs in Vδ1, Vδ2 T cells and CD4^+^T cells among HCs and PLWAHs at different timepoints. **(E, F)** Correlations of the frequencies of Vδ1Tregs with CD8^+^T cell activation at baseline and 96 weeks of ART were calculated by Spearman rank’s correlation test. *P < 0.05; ***P < 0.001; ****P < 0.0001.

γδ T cell subsets that express Treg-specific transcription factor Foxp3 are termed as γδ regulatory T cells (γδ Tregs), and are present at low frequencies in human peripheral blood (17, 18). However, the dynamics of the frequencies of γδ Tregs during the early stage of HIV-1 infection remain to be elucidated. Herein, we defined γδ Tregs as CD127^-^Foxp3^+^ cells, given their reduced expression of CD127. We initially compared the frequencies of Treg cells among CD4^+^T cells, Vδ1 and Vδ2 T cells in HCs and PLWAHs at baseline. We found that the frequencies of CD127^-^Foxp3^+^Vδ1 T cells were significantly higher compared to those of CD127^-^Foxp3^+^CD4^+^ T cells and CD127^-^Foxp3^+^Vδ2 T cells in both HCs and PLWAHs (**Figure 5A**), indicating that Vδ1 T cells are a predominant component of regulatory T cells.

Subsequently, we observed a significant reduction in the frequencies of CD127^-^Foxp3^+^Vδ1 T cells in PLWAHs at baseline compared to HCs, with this decrease persisting even after 96 weeks of early ART (**Figure 5B**). In contrast, the frequencies of CD127^-^Foxp3^+^CD4^+^T cells and CD127^-^Foxp3^+^Vδ2 T cells did not exhibit statistically significant changes in PLWAHs at baseline or at various time points post-ART initiation (**Figures 5C, D**). Furthermore, we analyzed the correlation between γδ Tregs and the activation levels of CD8^+^T cells. A negative association was observed between the frequencies of CD127^-^Foxp3^+^Vδ1 T cells and the frequencies of CD38^+^HLA-DR^+^CD8^+^T cells (*r* = -0.402, *P* = 0.009) after 96 weeks of early ART (**Figure 5F**), implying that γδ Tregs may suppress the activation of CD8^+^T cells. However, this correlation was not observed in PLWAHs at baseline (**Figure 5E**).

### γδ T cells mediate immunosuppression through TGF-β secretion

**Figure 6:**
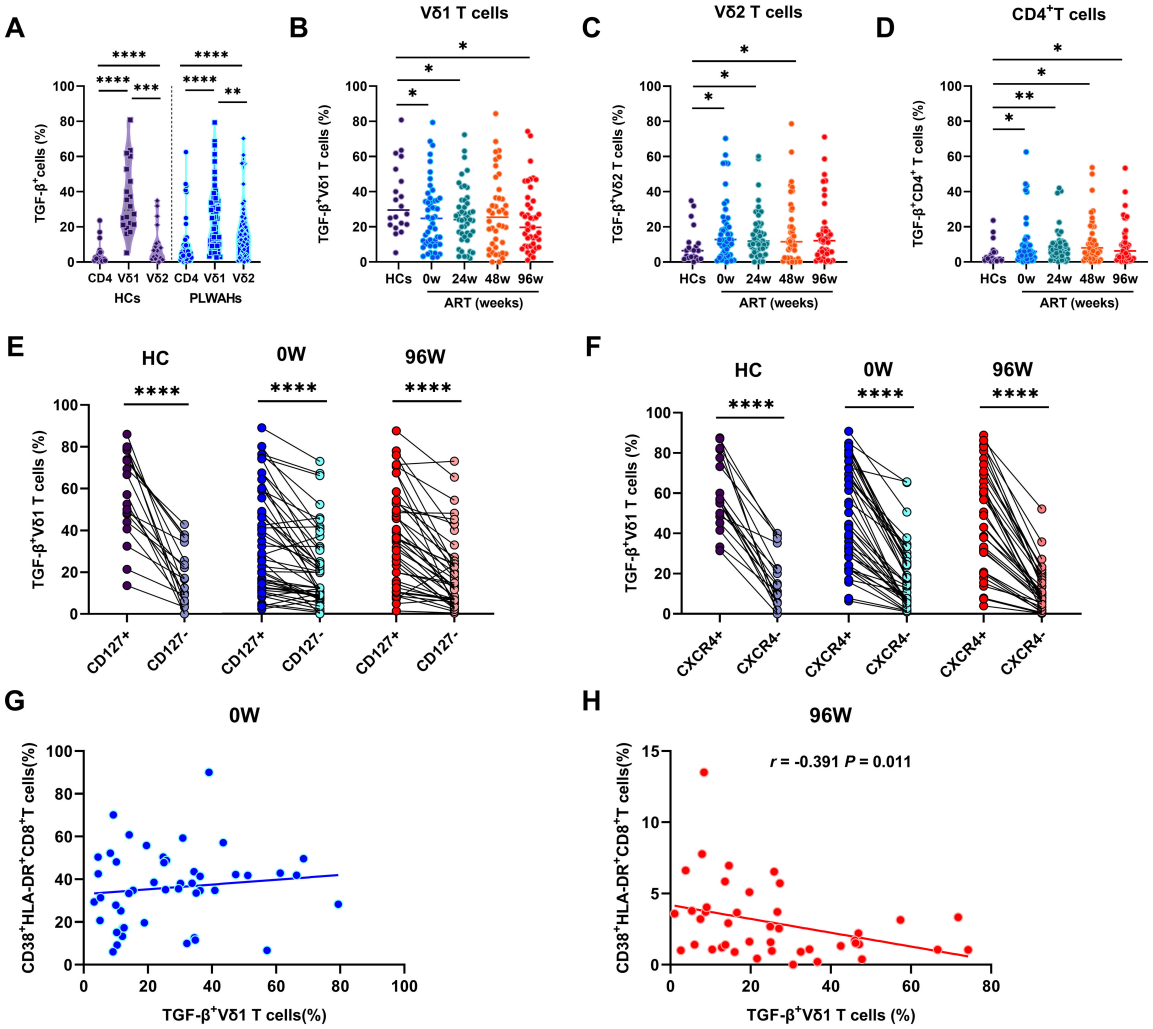
γδ T cells mediate immunosuppression through TGF-β secretion. **(A)** Comparison of the TGF-β secretion in CD4^+^T cells, Vδ1 and Vδ2 T cells in HCs and PLWAHs. Comparison of TGF-β expression in Vδ1 T cells **(B)**, Vδ2 T cells **(C)**, and CD4^+^T cells **(D)** among HCs and PLWAHs at different timepoints. **(E, F)** The expression of TGF-β in Vδ1 T cells were co-expressed with CD127 and CXCR4 in both HCs and PLWAHs. **(G, H)** Correlations of the frequencies of TGF-β^+^ Vδ1 T cells with CD8^+^T cell activation at baseline or 96 weeks of ART were calculated by Spearman rank’s correlation test. *P < 0.05; **P < 0.01; ***P < 0.001; ****P < 0.0001.

γδ T cells exert their suppressive effects through the secretion of cytokines and the expression of multiple inhibitory receptors. TGF-β is a critical pleiotropic cytokine involved in induction and maintenance of Tregs, and plays a pivotal role in modulating cellular processes, including the regulation of cell proliferation, differentiation, apoptosis, and immune function (19). In this study, we quantified TGF-β expression and found a significant upregulation in γδ T cell subsets, particularly within the Vδ1 subset, compared to CD4^+^ T cells (**Figure 6A**). This elevation was observed in both HCs and PLWAHs, indicating that γδ T cells are a major source of TGF-β secretion.

We further observed a significant decrease in the frequencies of TGF-β^+^Vδ1 T cells in PLWAHs at baseline and various time points post-ART compared to HCs (**Figure 6B**). Conversely, the frequencies of TGF-β^+^Vδ2 T cells and TGF-β^+^CD4^+^T cells were found to be statistically elevated in PLWAHs both pre- and post-ART at different time points, relative to HCs (**Figures 6C, D**). Additionally, Vδ1 T cells secreting TGF-β exhibited higher levels of co-expression of CD127 and the chemokine receptor CXCR4 (**Figures 6E, F**). Furthermore, we identified a negative correlation between the frequencies of TGF-β^+^Vδ1 T cells and those of CD38^+^HLA-DR^+^CD8^+^T cells after 96 weeks of ART (*r* = -0.391, *P* = 0.011), whereas no such association was observed at baseline (**Figures 6G, H**).

### Comparisons of the immune phenotypes between γδ T cell and CD4^+^ T cell

**Figure 7:**
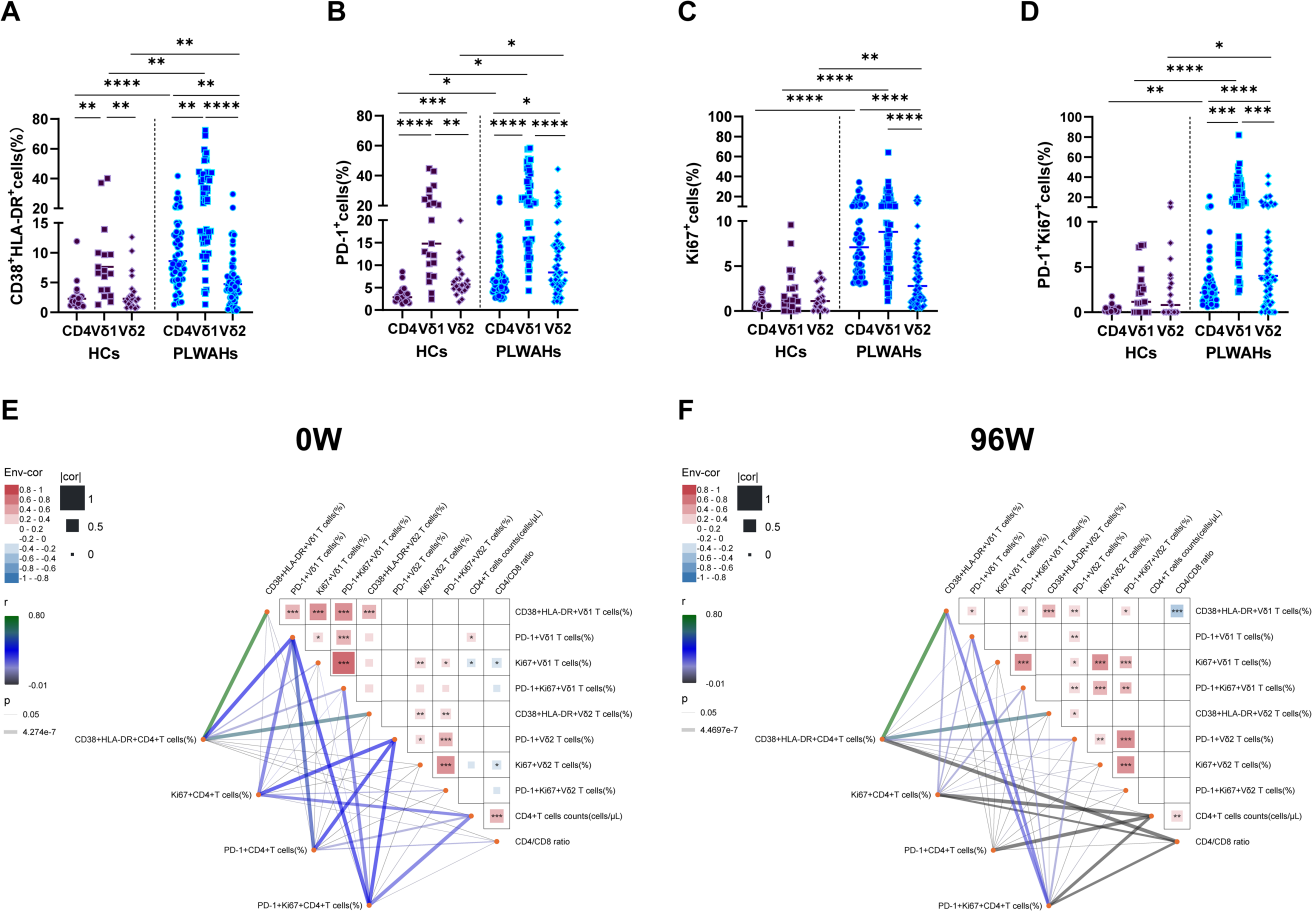
Comparison of immune phenotypes between γδ T cells and CD4^+^T cells. **(A-D)** Frequencies of CD38^+^HLA-DR^+^, PD-1^+^, Ki67^+^ and PD-1^+^Ki67^+^ cells among CD4^+^T cells, Vδ1 T cells and Vδ2 T cells were compared between HCs and PLWAHs. **(E, F)** Correlations of the frequencies of CD38^+^HLA-DR^+^, PD-1^+^, Ki67^+^ and PD-1^+^Ki67^+^ cells, as well as CD4^+^T cell counts and CD4/CD8 ratios, between γδ T cells and CD4^+^T cells at baseline **(E)** and after 96 weeks of ART **(F)**. Correlation coefficients were calculated using Spearman rank’s correlation test. *P < 0.05; **P < 0.01; ***P < 0.001; ****P < 0.0001.

To elucidate the role of γδ T cells in acute HIV infection, we compare the expression of activation and exhaustion markers between γδ T cell and CD4^+^ T cells. Our results demonstrated that the frequencies of CD38^+^HLA-DR^+^Vδ1 T cells were significantly higher than those of CD38^+^HLA-DR^+^CD4^+^ T cells and CD38^+^HLA-DR^+^Vδ2 T cells in both HC and PLWAH groups (**Figures 7A, B**). This suggests that Vδ1 T cells are intrinsically in a higher state of activation and are more readily activated. Furthermore, in HCs, no significant differences in Ki67 expression were observed among CD4^+^ T cells, Vδ1 T cells and Vδ2 T cells, irrespective of PD-1 expression. However, in PLWAH, the frequencies of Ki67^+^ and PD-1^+^Ki67^+^ cells were significantly elevated in CD4^+^ T cells, Vδ1 T cells and Vδ2 T cells compared to HCs. Notably, Vδ1 T cells exhibited the highest frequencies of Ki67^+^ and PD-1^+^Ki67^+^ cells compared to both CD4^+^ T cells and Vδ2 T cells (**Figures 7C, D**).

Subsequently, we examined the correlations between the expression of activation exhaustion and proliferation markers in CD4^+^ T cells, Vδ1 T cells and Vδ2 T cells, as well as their associations with CD4^+^ T cell counts and CD4/CD8 ratios at baseline and after 96 weeks of ART. At baseline, the frequencies of PD-1^+^ Vδ1 T cells exhibited a positive correlation with CD4^+^ T cell counts. In contrast, the frequencies of Ki67^+^Vδ1 T cells and Ki67^+^Vδ2 T cells showed negative correlations with CD4^+^ T cell counts and CD4/CD8 ratios, respectively. Additionally, after 96 weeks of ART, the frequencies of CD38^+^HLA-DR^+^Vδ1 T cells were negatively associated with CD4/CD8 ratios (**Figures 7E, F**).

Furthermore, we observed that the frequencies of PD-1^+^CD4^+^ T cells, Ki67^+^ CD4^+^ T cells, and PD-1^+^Ki67^+^CD4^+^ T cells were positively correlated with CD4^+^ T cell counts at baseline, but displayed negative associations with CD4^+^ T cell counts or CD4/CD8 ratios after 96 weeks of ART. Importantly, the expression of CD38, HLA-DR, PD-1 and Ki67 among CD4^+^ T cells, Vδ1 T cells and Vδ2 T cells formed a highly interconnected network at both baseline and 96 weeks of ART (**Figures 7E, F**). Collectively, these findings suggest that the observed correlations in immune markers expression reflect functional interactions between the γδ T cells and CD4^+^ T cells.

## Discussion

The current study provides a detailed analysis of the immunological dynamics of γδ T cell subsets in individuals with acute HIV-1 infection following early initiation of ART. For the first time, we conducted a simultaneous comparative analysis of the phenotypic and functional markers across both CD4^+^ T cells and γδ T cell subsets. This approach has revealed the complex interplay between γδ T cell dynamics and HIV-1 disease progression, while providing insights into the role of γδ T cells frequency and function alterations in immune reconstitution. Our findings offer novel perspectives for developing γδ T cell-based immunotherapeutic strategies, which should be specifically designed to target either Vδ1 or Vδ2 T cell subsets based on their distinct phenotypic and functional characteristics.

Early ART initiation is crucial for increasing CD4^+^ T cell counts and improving CD4/CD8 ratios, as presented in **Table 1**. Our study firstly reported that early ART significantly boosts Vδ2 T cells frequencies at various time points, a benefit that not seen with late-stage ART initiation (20). However, Vδ1 T cell frequencies and Vδ1/Vδ2 ratios remain high even after ART (**Figure 1**), suggesting that early ART may not fully restore γδ T cell subset balance. Prior findings link persistent innate inflammatory cytokines, even post-early ART, to Vδ1 and Vδ2 T cells during HIV-1 infection (21, 22), potentially causing γδ T cell immunity imbalance in PLWHs due to chronic and persistent inflammation.

The mechanisms governing the numeric expansion of Vδ1 T cells and the inversion of the Vδ1/Vδ2 ratio during HIV-1 infection remain unclear. Current evidence suggests a multifactorial process involving several key mechanisms: First, HIVgp120 interacts with CCR5, which is highly expressed on Vδ2 T cells, leading to the activation of the p38 and MAPK pathways and inducing Vδ2 T cell apoptosis (23). The depletion of gut Vδ2 T cells compromises the intestinal epithelial barrier integrity, resulting in microbial translocation, and the development of a systemic pro-inflammatory milieu that promotes Vδ1 T cell expansion (24). Second, the upregulation of CD4 on activated Vδ2 T cells facilitates HIV-1 entry into these cells, contributing to the establishment of HIV-1 latency (25). Third, the cytotoxic activity of NKG2C^+^Vδ1 T cells eliminate HIV-1-infected CD4^+^T cells and suppress HIV-1 replication (16, 26), may also contribute to Vδ2 T cell reduction.

The correlation analyses suggesting that γδ T cells may play dual role in HIV-1 pathogenesis: they may exert protective effects during early infection but potentially contribute to disease progression through mechanisms that exacerbate pathological processes or impede immune recovery. The genetic profiles of Vδ1 T cells and Vδ2 T cells in healthy donors are markedly distinct (27). Therefore, advanced technologies, such as single-cell RNA sequencing, proteomics and mass cytometry are needed to further elucidate the phenotypic and functional characteristics of γδ T cell subsets and their roles in HIV-1 pathogenesis.

T cell dysfunction, characterized by PD-1 expression and heightened proliferation, is key to HIV-1 disease progression and hinders immune reconstitution (3, 28–30). Though early ART can normalize T cell activation, it fails to curb proliferation, particularly within PD-1^+^ T cells in acute HIV-1 infection (7). The current study reveals that early ART lowers activated γδ T cells frequency in PLWAHs, but Ki67 expression, especially within the PD-1^+^ subsets, persists elevated (**Figures 2**, **3**). This suggests a continuous state of immune exhaustion, likely driven by persistent inflammation, and highlights the necessity for therapeutic interventions targeting PD-1 in acute HIV-1 infection (31, 32).

Moreover, elevated circulating T cell proliferation represents a characteristic feature of cellular senescence and aging. The accumulation of cytoplasmic DNA in senescent cells triggers the activation of Akt and mTOR pathways, which in turn drive CD4^+^T cell proliferation and activation (33). Senescence-associated T cells, a subset of CD4^+^T cell with a PD-1 memory phenotype, exhibit impaired responsiveness to TCR stimulation, but actively produce inflammatory cytokines (34). Consequently, the persistence of PD-1^+^Ki67^+^ T cells may contribute to chronic inflammation and disrupt T cell homeostasis. To further advance our understanding, future in-depth investigation employing multi-omics approaches will be essential to delineate the precise phenotypic characteristics and regulatory networks governing PD-1^+^Ki67^+^ T cells dynamics. Such comprehensive analyses are expected to provide critical insights into the molecular mechanisms underlying immune dysregulation in HIV-1 infection.

PD-1 and CD56 are markers for accessing the cytotoxicity of γδ T cells in umbilical cord blood. PD-1^-^CD56^+^ Vδ2 T cells exhibit increased expression of perforin and cytotoxicity genes (35). Our study found elevated perforin expression in PD-1^-^ and CD56^+^ Vδ1 and Vδ2 T cells in PLWAHs, sustained high even after ART (**Figure 4**). Furthermore, Vδ2 T cells have lower perforin than Vδ1 T cells, and this disparity is not mitigated by early ART initiation. Given both subsets can lyse HIV-1-infected CD4^+^ T cells (16, 36), γδ T cells may play a role in HIV-1 control and latency elimination. Further research is needed to uncover the intricate and detailed mechanisms by which γδ T cell subsets influence HIV-1 infection and respond to ART.

γδ T cells, especially Vδ1, are known for regulatory roles through Foxp3 and TGF-β production (17, 18, 37, 38). While Vδ2 T cells, which produce less TGF-β, modulate T cell activation in primary HIV-1 infection (39). Our study uniquely assesses Foxp3 and TGF-β in CD4^+^, Vδ1 and Vδ2 T cells across HCs and PLWAHs. We found Vδ1 T cells have the highest Foxp3 and TGF-β (**Figures 5**, **6**), highlighting their regulatory dominance. Additionally, we linked phenotypes with Foxp3 and TGF-β expression and observed that CD127^-^ cells showed increased Foxp3, while CD127^+^ cells had elevated TGF-β. Moreover, TGF-β^+^ cells co-expressed CXCR4 (**Figure 6**). PD-1’s expression on Treg cells correlates with Foxp3 expression (40, 41), but its role in γδ Tregs is unclear and requires further investigation.

A sustained and robust HIV-1-specific CD8^+^ T cell immune response is crucial for controlling HIV-1 infection in post-treatment controllers, who initiated ART during acute infection (42, 43). These individuals exhibit lower levels of CD8^+^ T cell activation and exhaustion, yet the extent of CD8^+^ T cell proliferation remains unassessed. Echoing these findings, our previous work indicated that early initiation of ART can mitigate T cell activation but has a limited effect on proliferation (7). Moreover, we have noted negative correlations between the frequencies of CD127^-^Foxp3^+^ or TGF-β^+^ Vδ1 T cells and CD8^+^ T cell activation, which points to a potential immunosuppressive function in regulating CD8^+^ T cell responses. Therefore, further investigation into the precise role and mechanisms of Vδ1Treg cells, utilizing both ex vivo experimental systems and animal models, may provide critical insights for the design and development of novel immunotherapeutic strategies.

## Conclusion

In summary, our study has unveiled the dynamic immunological characteristics of γδ T cell subsets in PLWAHs following early ART initiation. We found that early ART does not fully restore γδ T cell numbers, phenotypes, and functions. These insights offer a foundation for the development of immunotherapeutic strategies aimed at γδ T cells to promote immune reconstitution. However, our research has its constraints. Firstly, the sample size was limited, and the study included only male participants. Secondly, we assessed the phenotypes and functions of γδ T cells using a select set of markers; a broader array of markers is required to more accurately and comprehensively understand the precise role of γδ T cells in future studies.

## Data Availability

The raw data supporting the conclusions of this article will be made available by the authors, without undue reservation.
